# Editorial: Mycotoxins in pig feed: health risks, reproductive impacts, and diagnostic innovations

**DOI:** 10.3389/fvets.2025.1675111

**Published:** 2025-09-11

**Authors:** Panagiotis Tassis, Alix Pierron, Johannes Kauffold

**Affiliations:** ^1^Clinic of Farm Animals, Faculty of Health Sciences, School of Veterinary Medicine, Aristotle University of Thessaloniki, Thessaloniki, Greece; ^2^IALTA, IHAP INRAE-ENVT, Université de Toulouse, Toulouse, France; ^3^Clinic for Ruminants and Swine, Faculty of Veterinary Medicine, University of Leipzig, Leipzig, Germany

**Keywords:** mycotoxins, pig health, deoxynivalenol (DON), zearalenone (ZEN), ochratoxin A, pig reproduction, detoxification agent

Mycotoxins, as secondary metabolites of fungi such as *Fusarium, Aspergillus*, and *Penicillium* are detected in grains, globally and remain one of the most insidious threats in modern swine production systems. The Research Topic presented in Frontiers in Veterinary Science Journal focused on research findings of a part of the mycotoxins issue in pig production including toxicological mechanisms, reproductive outcomes, feed mitigation strategies, and diagnostic advancements. Four articles providing interesting scientific results were included in the Research Topic.

In the article by Qin et al., the positive effects of selenized-oligochitosan (SOC) were demonstrated against intestinal dysfunction induced by ingestion of significant zearalenone (ZEN) levels for 42 days in piglets. The selenized-oligochitosan administered at the highest dosage level (0.5 μg/g SOC), resulted in significant reduction of intestinal dysfunction biomarkers, as well as in improvement of the digestive enzymes activity and the intestinal barrier integrity.

Further, the report by Tassis et al. covered aspects of health and performance improvements observed after the introduction of a novel multi-component detoxifying agent (MMDA) against combined ZEN and ochratoxin A (OTA) exposure in weaned pigs. The effects of the novel detoxifying agent were highlighted at the greatest dosage level (3 g MMDA/kg feed) tested with emphasis on improved feed conversion ratio (FCR) and reduction of mycotoxins residues in tissues.

The article of Benthem de Grave et al. highlighted the effects of an algoclay-based mycotoxin decontaminant in sows and their progeny in deoxynivalenol (DON) and ZEN exposed sows. The study was performed with combined dietary exposure of ~100 or 300 μg ZEN/kg feed and 250 μg DON/kg feed in sows during the last week of gestation and the lactation period (26 days). The test substance was able to decrease the levels of ZEN and its metabolites in the serum of sows and decrease de-epoxy-DON in the serum of piglets, thus providing evidence of reduced ZEN intestinal absorption and subsequently reduced exposure of piglets.

Moreover, data on the effects of a 42-day low DON-exposure in pre-pubertal gilts on the immunohistochemical expression of estrogen receptors and expression of genes encoding selected large intestine enzymes associated with various metabolic and detoxification processes, was presented by Gajecka et al.. The modulation of α and β estrogen receptors, as well as GSTP1 mRNA and CYP1A1 mRNA expression in various intestinal segments, was highlighted, whereas a probable protection offered by DON (at the dosage level and time period used in the study) against uncontrolled proliferation in the large intestine of pre-pubertal gilts was demonstrated.

Taken together the above-mentioned contributions in the Research Topic highlighted significant aspects of gene alterations, enzyme and estrogenic receptor modulation, variability of the bioavailability of particular mycotoxins after dietary exposure, either alone or in combination to pigs, as well as possible mechanisms of action against the detrimental effects of mycotoxins *in vivo*. The presence and effects of mycotoxins combinations has been emphasized by several contributions reporting that even low-level or subclinical exposure to mycotoxins such as DON, ZEN and OTA, can compromise pig health and performance (1–3). In breeding herds, the estrogenic effects of ZEN are particularly concerning (4). Field and laboratory evidence suggest persistent effects on sow fertility, placental function, and fetal development (5, 6). Together, these findings challenge traditional safety thresholds and suggest the need for regulatory frameworks that consider cumulative and synergistic mycotoxin exposures.

Among mitigation approaches, multicomponent mycotoxin detoxifying agents (MMDAs) yield special attention. Raj et al. (7) demonstrated the *in vivo* efficacy of a composite additive—comprising of modified zeolite, Bacillus spores, yeast cell wall, and silymarin, in reducing tissue residues of ZEN and T-2 toxin in piglets, whereas in another *in vivo* trial with the same multicomponent agent, its ability to improve the average daily gain and FCR, and reduce DON residues in kidneys of weaned pigs receiving contaminated feed with DON and ZEN, was reported (8). Moreover, Papatsiros et al. (9) demonstrated beneficial effects of another multi-component mycotoxin-detoxifying agent, containing clays (bentonite, sepiolite), phytogenic feed additives (curcumin, silymarin) and post-biotics (yeast cell wall, hydrolyzed yeast), with reduction of oxidative stress biomarkers and improvement of health parameters in lactating sows. Similarly, novel solutions such as the use of lithocholic acid (LCA) against DON-induced detrimental effects are also emerging (10). The latter, modulates DON-induced inflammation in intestinal epithelial cells via epigenetic mechanisms, opening the door for functional feed additives that act at the molecular level (11).

In conclusion, the editorial board extends its gratitude to all contributing authors and reviewers of the Research Topic. The comprehensive and multidisciplinary approaches showcased here reinforce the urgent need for integrated solutions that safeguard swine health and performance. Articles in the present Research Topic highlight particular aspects of mycotoxins effects and present effective promising solutions which could tackle the various detrimental effects of combined mycotoxins exposure in pigs. As observed frequently, the occurrence of such combined exposure is a common pattern in pig feed worldwide (12), therefore the need for targeted research efforts on mycotoxins interactions *in vivo*, as well as on effective mitigation strategies against combined exposure should be a focus point for further research efforts. Therefore, future studies should focus also on the development of possible multi-mycotoxin safety limits based on additive and synergistic effects when mycotoxin mixtures are present in pig feed and the integration of regular analytical diagnostics and feed mitigation strategies at farm level through the support of field-deployable technologies.
